# Pharmacological Effects of Salvianolic Acid B Against Oxidative Damage

**DOI:** 10.3389/fphar.2020.572373

**Published:** 2020-11-11

**Authors:** Zhun Xiao, Wei Liu, Yong-ping Mu, Hua Zhang, Xiao-ning Wang, Chang-qing Zhao, Jia-mei Chen, Ping Liu

**Affiliations:** ^1^ Institute of Interdisciplinary Medicine, Shanghai University of Traditional Chinese Medicine, Shanghai, China; ^2^ Institute of Liver Diseases, Key Laboratory of Liver and Kidney Diseases (Ministry of Education), Shuguang Hospital Affiliated to Shanghai University of Traditional Chinese Medicine, Shanghai, China; ^3^ Shanghai Key Laboratory of Traditional Chinese Medicine, Shanghai, China

**Keywords:** salvianolic acid B, oxidative damage, ROS, Nrf2, Keap1

## Abstract

Salvianolic acid B (Sal B) is one of the main active ingredients of *Salvia miltiorrhiza*, with strong antioxidant effects. Recent findings have shown that Sal B has anti-inflammatory, anti-apoptotic, anti-fibrotic effects and can promote stem cell proliferation and differentiation, and has a beneficial effect on cardiovascular and cerebrovascular diseases, aging, and liver fibrosis. Reactive oxygen species (ROS) include oxygen free radicals and oxygen-containing non-free radicals. ROS can regulate cell proliferation, survival, death and differentiation to regulate inflammation, and immunity, while Sal B can scavenge oxygen free radicals by providing hydrogen atoms and reduce the production of oxygen free radicals and oxygen-containing non-radicals by regulating the expression of antioxidant enzymes. The many pharmacological effects of Sal B may be closely related to its elimination and inhibition of ROS generation, and Nuclear factor E2-related factor 2/Kelch-like ECH-related protein 1 may be the core link in its regulation of the expression of antioxidant enzyme to exert its antioxidant effect. What is confusing and interesting is that Sal B exhibits the opposite mechanisms in tumors. To clarify the specific target of Sal B and the correlation between its regulation of oxidative stress and energy metabolism homeostasis will help to further understand its role in different pathological conditions, and provide a scientific basis for its further clinical application and new drug development. Although Sal B has broad prospects in clinical application due to its extensive pharmacological effects, the low bioavailability is a serious obstacle to further improving its efficacy *in vivo* and promoting clinical application. Therefore, how to improve the availability of Sal B *in vivo* requires the joint efforts of many interdisciplinary subjects.

## Introduction

**FIGURE 1:**
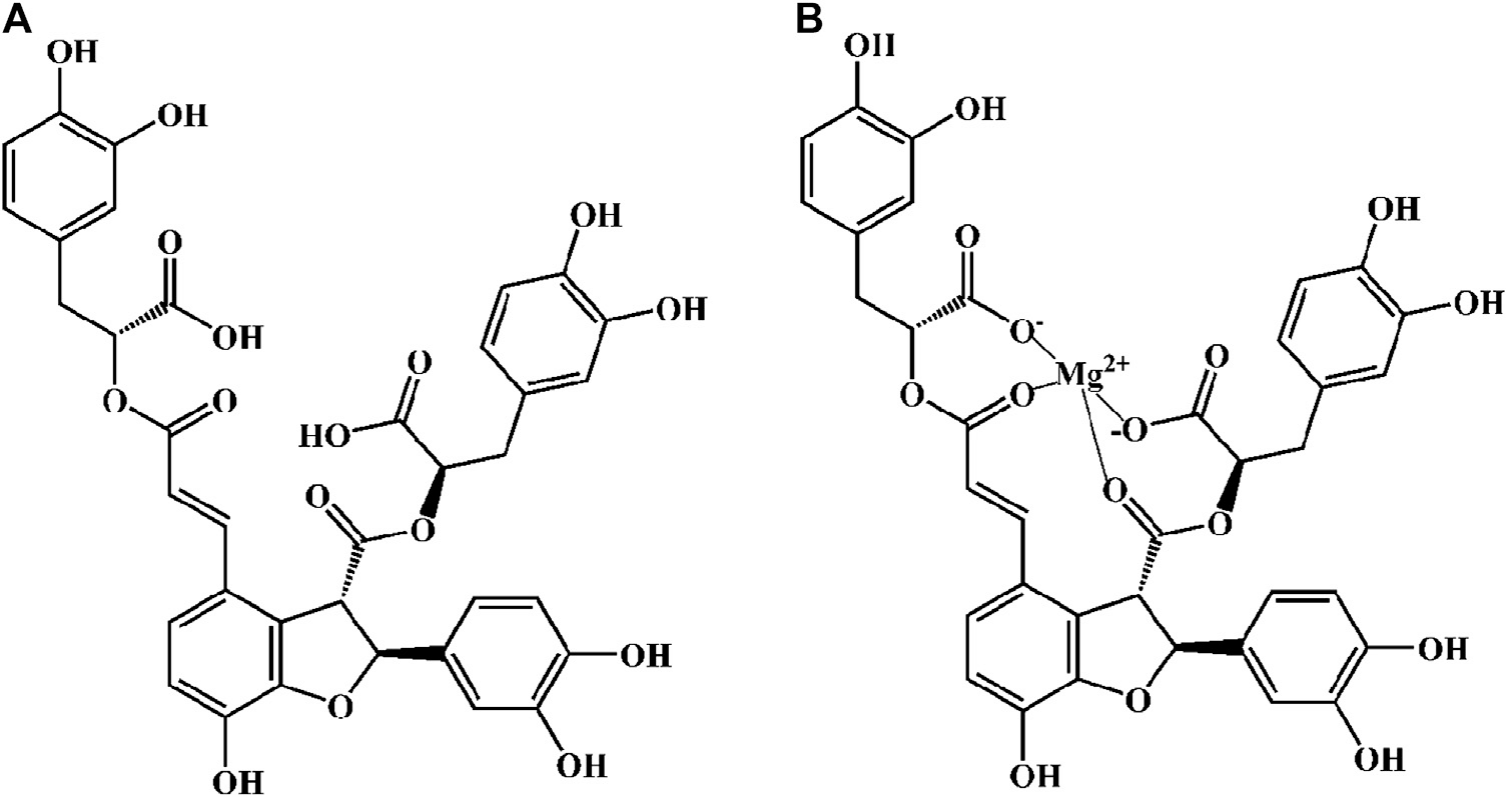
The molecular structural of salvianolic acid B (Sal B) and magnesium tanshinoate B (MTB). **(A)** Sal B, PubChem substance SID: 6451084; **(B)** MTB, PubChem substance SID: 13507533.

**FIGURE 2:**
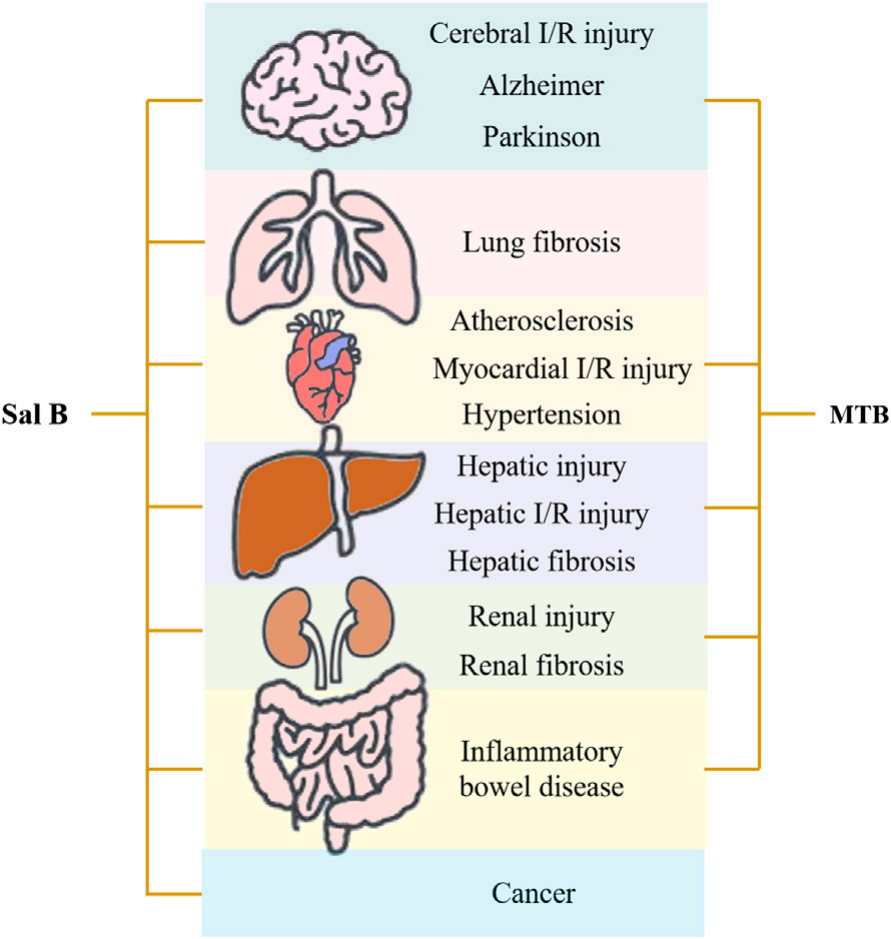
Pharmacological effects of salvianolic acid B (Sal B) and magnesium tanshinoate B (MTB).

Danshen, *Radix Salviae miltiorrhizae*, is the dry root and rhizome of *Salvia miltiorrhiza* Bge. Its medicinal ingredients are mainly lipophilic diterpene quinones and hydrophilic phenolic acids, as well as flavonoids, triterpenes, and sterols. Salvianolic acid B (Sal B) is also named lithospermic acid B. It is one of the most abundant and active ingredients in the hydrophilic components of *S. miltiorrhiza*. Sal B is synthesized by the condensation of three molecules of 3,4-dihydroxyphenyllactic acid and one molecule of caffeic acid. Its molecular formula is C_36_H_30_O_16_, and its molecular weight is 718. Its magnesium salt, magnesium tanshinoate B (MTB) is mostly used and studied (Figure 1). It has been shown that Sal B has antioxidant, antiinflammatory, and anti-fibrotic effects, and inhibits apoptosis (Cao et al., 2012), while MTB has similar pharmacological effects (Wu and Wang, 2012; Song et al., 2014; Lin et al., 2019). Both of them have important effects on various organs such as the heart, brain, liver, kidney, and intestines (Figure 2). As magnesium ions have important physiological effects on the brain, heart and skeletal muscles (de Baaij et al., 2015), MTB may have a better effect than Sal B on cardiovascular and cerebrovascular diseases, such as cerebral infarction, myocardial infarction and coronary atherosclerosis. Even other diseases such as aging, hepatic fibrosis and tumors are closely related to abnormal blood microcirculation. Therefore, MTB may have a better curative effect than Sal B on the diseases with abnormal blood circulation. The strong antioxidant effects may be one of the basis of other pharmacological effects of Sal B and MTB. In view of the fact that the current research on Sal B is more extensive and comprehensive than MTB, this article reviews the research progress of pharmacological effects of Sal B in recent years based on its antioxidant effects.

## Redox Balance and Oxidative Damage

Redox reactions are involved in almost all life processes. Oxidative stress at physiological status helps regulate life processes, but excess oxidants can induce cell damage and even death. However, even in different organelles of the same cell, redox homeostasis may be different (Jones and Sies, 2015), which explains why peroxidation has different degrees of damage to different organelles or cells. The nicotinamide adenine dinucleotide (NADH) system integrates catabolism and energy capture, while the NADH phosphate (NADPH) system not only drives reductive anabolic metabolism but also drives oxidation reactions controlled by enzymes such as NADPH oxidase (NOX) and nitric oxide synthase (NOS) (Sies et al., 2017).

**FIGURE 3:**
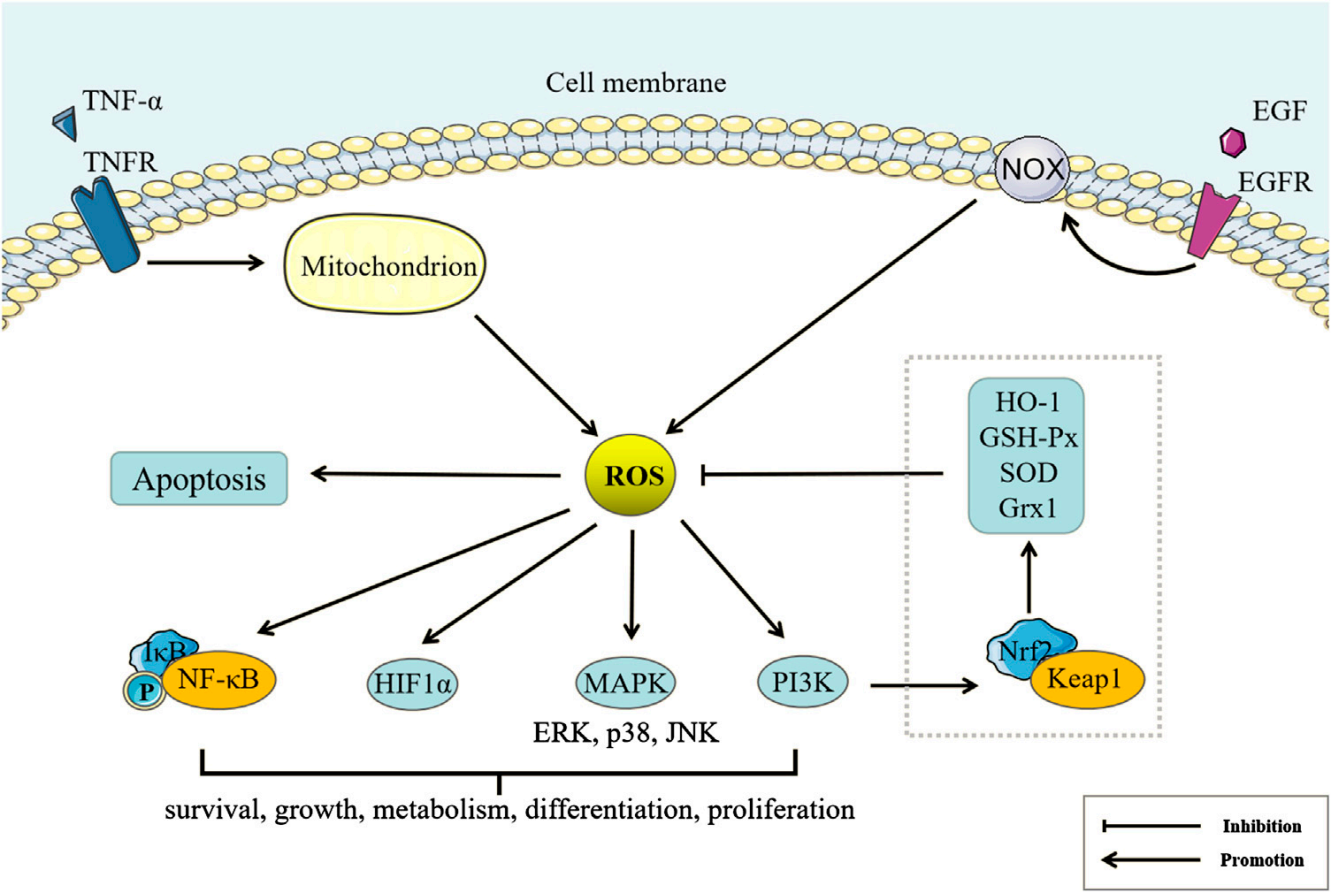
Reactive oxygen species (ROS) and Peroxidation Damage. Tumor necrosis factor (TNF)-α and EGF can regulate ROS production of mitochondria and NADPH oxidase (NOX)-catalyze by binding the corresponding membrane receptors. Under physiological conditions, ROS can also regulate Nuclear factor E2-related factor 2/Kelch-like ECH-related protein 1 through the phosphatidylinositol 3-kinase pathway to increase the expression of antioxidant enzymes, inhibit ROS accumulation, and maintain redox balance. Under pathological conditions, excess ROS can regulate cell survival, growth, metabolism, differentiation, proliferation, and apoptosis by Nuclear factor κB (NF-κB), HIF1α, Mitogen-activated protein kinase (MAPK), phosphatidylinositol 3-kinase and other pathways.

NOX, NOS, and cyclooxygenase (COX) can produce reactive oxygen species (ROS), such as a single electron reduction product of oxygen, including superoxide anion radical (${\text{O}}_{2}^{-}\text{·}$), hydroxyl radical (OH·) and other oxygen-containing free radicals, as well as non-radical oxygen-containing molecules such as hydrogen peroxide (H_2_O_2_) and singlet oxygen (^1^O_2_) (Holmström and Finkel, 2014). ROS can oxidize polyunsaturated fatty acids to peroxidized fatty acids, which can rearrange and further react to form a large variety of secondary oxidation products, such as malondialdehyde (MDA). Both oxygen-containing free radicals and lipid peroxidation product MDA can cause the cross-linking of membrane protein molecules (Moldogazieva et al., 2019), while oxygen-containing non-radicals are mainly used as the second messengers to participate in intracellular signal transduction and regulation (Sies et al., 2017). It has been shown that ROS can mediate cell proliferation, survival, death, and differentiation and control inflammation, immunity, and tumors by inhibiting or activating proteins, promoting DNA mutation, and activating gene transcription (Nathan and Cunningham-Bussel, 2013). Therefore, the regulation of ROS can directly affect the fate of cells (Figure 3). For example, tumor necrosis factor α (TNF-α) can participate in the regulation of nuclear factor κB (NF-κB)-induced cell survival pathway and c-Jun terminal kinase (JNK)-induced cell death pathway through inducing mitochondrial ROS production (Blaser et al., 2016). It is worth noting that although the accumulation of ROS can also lead to cell autophagy, the role of ROS-induced autophagy in cell survival and death is still controversial, which may be related to the location and level of ROS in different environments (Scherz-Shouval and Elazar, 2011a). Nuclear factor E2-related factor 2 (Nrf2) is a regulator of cell resistance to oxidants, and can regulate the expression of a variety of antioxidant enzymes such as superoxide dismutase (SOD), glutathione peroxidase (GSH-Px), heme oxygenase-1 (HO-1), and gluredoxin 1 (Grx1) (Ma, 2013). Kelch-like ECH-related protein 1 (Keap1) can inhibit the transcriptional activity of Nrf2 under stress-free conditions through rapidly ubiquitinating and degrading Nrf2. Accumulation of ROS can trigger Keap1 proteolysis, which activates Nrf2 signaling and increases the expression of antioxidant enzymes (Yamamoto et al., 2018). Therefore, the Nrf2/Keap1 system is an important switch for oxidative stress regulation.

## Mechanisms of Salvianolic Acid B Against Oxidative Damage

**FIGURE 4:**
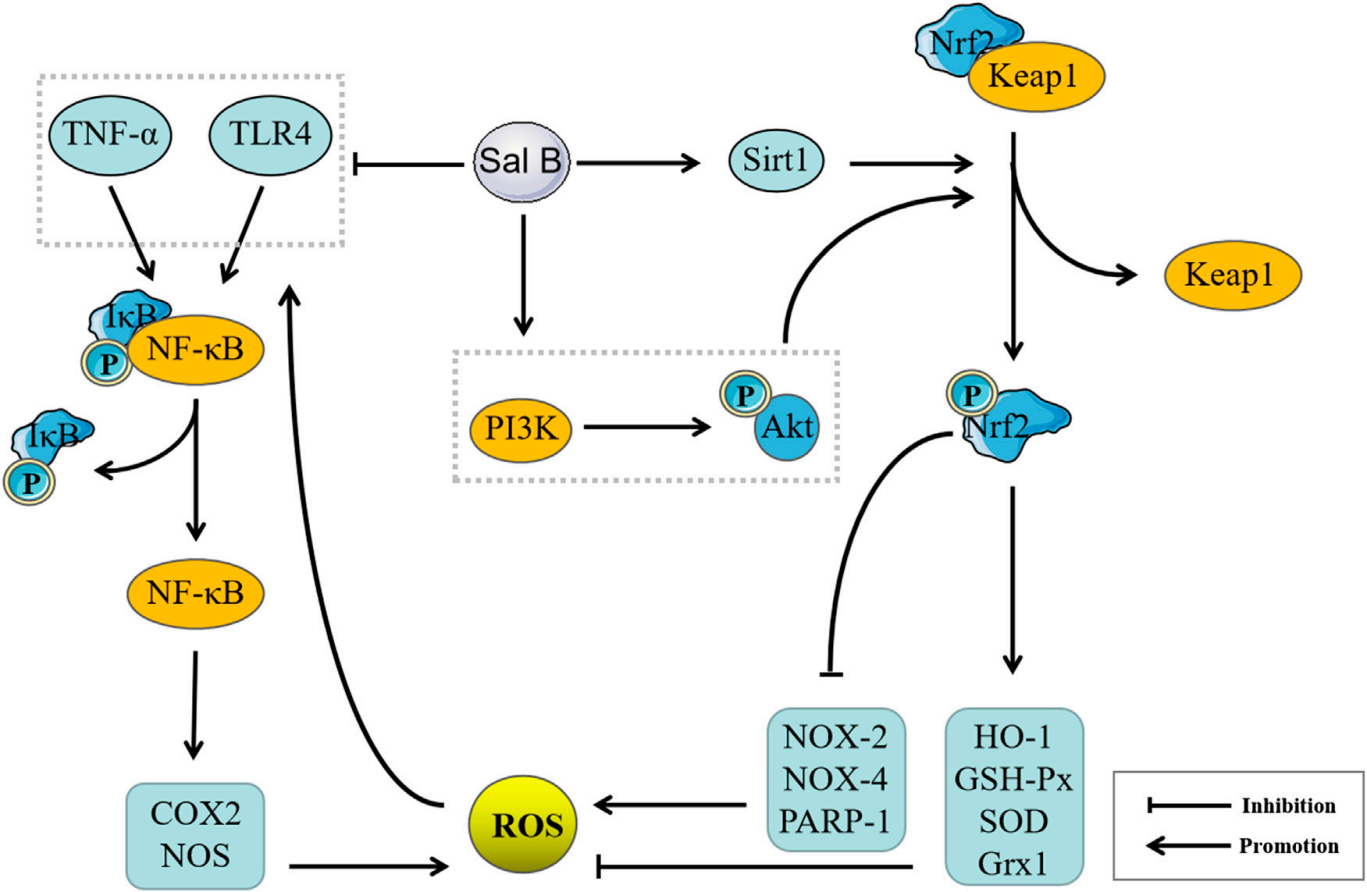
Pharmacological mechanisms of Salvianolic acid B (Sal B) based on antioxidant effect. Sal B can inhibit the expressions of Tumor necrosis factor (TNF)-α, Cyclooxygenase (COX2) and NADPH oxidase (NOS) by inhibiting TNF-α/Nuclear factor κB (NF-κB) and Toll-like receptor (TLR4)/NF-κ-B pathways, and promote the expression of antioxidant enzymes such as Heme oxygenase-1 (HO-1), NQOD, Superoxide dismutase (SOD) and inhibit the expression of oxidases such as NOX-2, NOX-4, Poly (ADP-ribozyme) polymerase 1 (PARP-1) by the Nuclear factor E2-related factor 2/Kelch-like ECH-related protein 1 pathway, and then inhibit Reactive oxygen species (ROS) generation, improve inflammation, cell apoptosis, autophagy, fibrosis, microcirculation disorders, and stem cell proliferation and differentiation.

As containing nine phenolic hydroxyls, Sal B can donate a number of hydrogen atoms to exert a powerful antioxidant effect, and previous studies have confirmed that Sal B has a strong free radical scavenging ability (Huang and Zhang, 1992; Lin et al., 2006; Chen C.-Y. et al., 2013). In addition to its own antioxidant properties, Sal B may also exert its antioxidant effects on other targets. This effect may be mainly reflected in the regulation of non-radical ROS. Sal B can regulate the expression of various antioxidant enzymes, such as increasing the expression of SOD, GSH-Px, and HO-1 (Lee et al., 2014; Zhao et al., 2017), inhibiting the expression of NOX-2 and NOX-4 (Ling et al., 2017), inhibiting the poly (ADP-ribozyme) polymerase 1 (PARP-1) activity to prevent NAD + depletion (Liu et al., 2014) and upregulating Grx1 expression (Liu et al., 2016), thereby effectively inhibiting the generation of ROS and reducing the production of lipid peroxidation products such as MDA, to exert an antioxidant effects (Yang et al., 2011). In addition, ROS also can activate NF-κB through Toll-like receptor (TLR)4 and TNF-α to promote the expressions of COX2 and NOS, and further promote the generation of ROS (Zhang et al., 2016). Sal B had inhibitory effects on TNF-α/NF-κB and TLR4/NF-κ-B signaling pathways (Wang et al., 2010; Wang et al., 2016). Sal B can down-regulate Keap1 and upregulate Nrf2 expression through NAD-dependent deacetylase Sirtuin 1 (SIRT1) and phosphatidylinositol 3-kinase (PI3K)/Akt signaling pathway (Tongqiang et al., 2016; Zhang et al., 2018), thereby promoting the production of antioxidant enzymes. Its regulation of TNF-α/NF-κB and TLR4/NF-κB signaling pathways may be achieved by regulating Nrf2/Keap1, therefore, regulation of Nrf2/Keap1 may be the core target of its anti-oxidation mechanism (Figure 4).

## Pharmacological Mechanisms of Salvianolic Acid B Based on Anti-Oxidation

### Anti-Oxidation and Anti-Inflammatory Effects of Salvianolic Acid B

**FIGURE 5:**
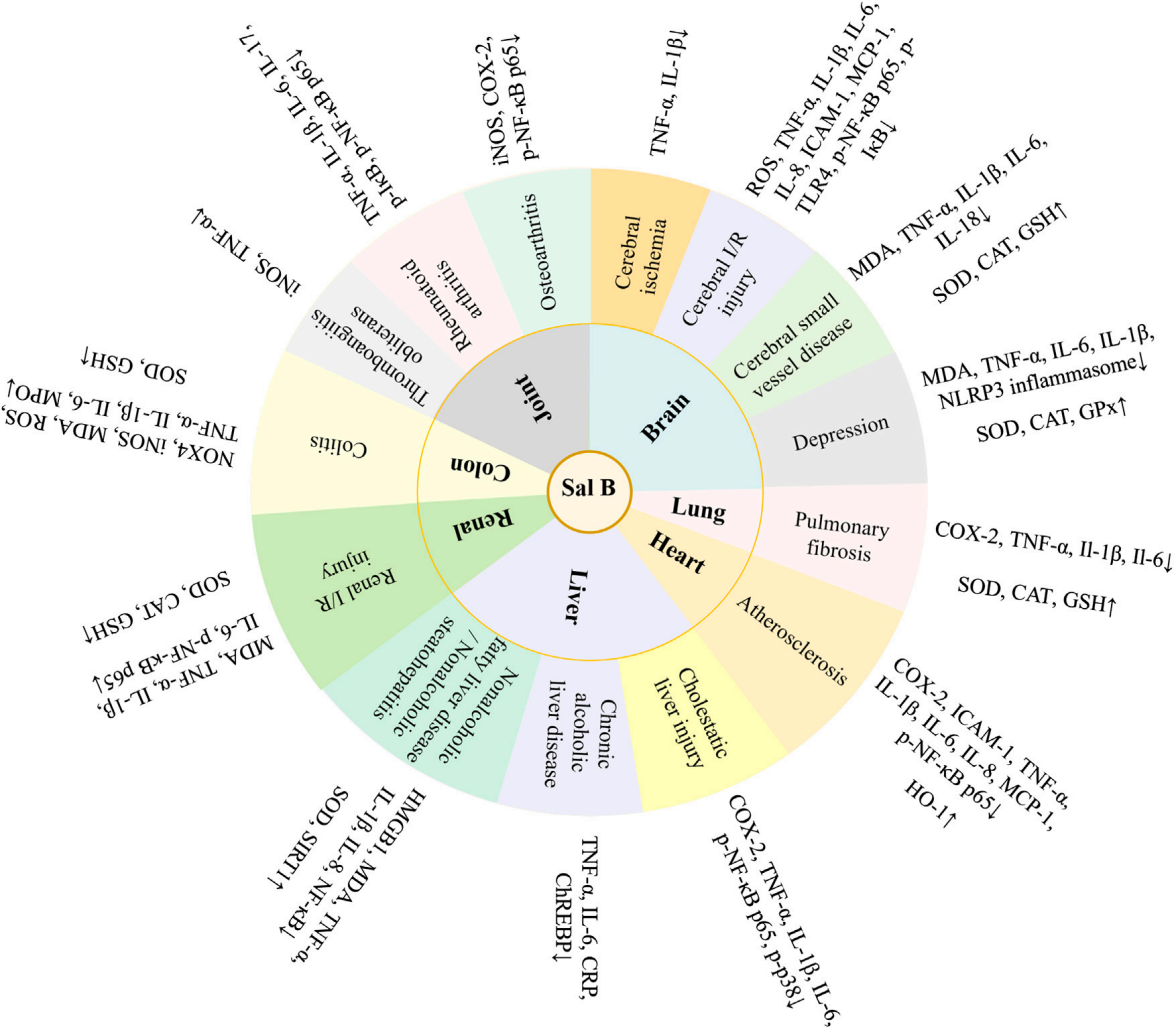
Mechanisms of Salvianolic acid B (Sal B) in different chronic inflammatory diseases. In addition to increasing the expression of antioxidant enzymes such as Superoxide dismutase (SOD), CAT, GSH, and reducing the expression of oxidative enzymes such as Cyclooxygenase (COX-2), NADPH oxidase (NOX)-4, iNOS to inhibit the generation of Reactive oxygen species (ROS) and malondialdehyde (MDA), Sal B has been shown to improve chronic inflammation in a variety of disease models, suggesting that its anti-inflammatory and antioxidant effects are closely related.

Inflammatory reaction is an important active defense system of the body. It can induce natural immune response cells to release inflammatory factors. Continuous or excessive secretion of inflammatory factors can damage normal tissues and cells. Recent evidence has suggested that oxidative stress plays a vital role in the development and persistence of inflammation, and oxidants can affect all stages of the inflammatory response (Lugrin et al., 2014). Due to the paradoxical role of antioxidant enzymes, it is unclear whether oxidative stress is the cause or the result of pathological conditions (Lei et al., 2016), however it is certain that they at least they coexist and affect each other. Low-density lipoprotein (LDL) can be oxidized by ROS to oxidized LDL (ox-LDL), which stimulates endothelial cells to secrete various inflammatory factors and induces monocyte adhesion and migration into the intima of the artery in atherosclerosis. Sal B can inhibit the production of ox-LDL (Sun et al., 2011), inhibit the aggregation of macrophages and reduce the uptake of ox-LDL by macrophages through antioxidant effects (Bao et al., 2012), indicating a correlation between the anti-inflammatory and antioxidant effects of Sal B. NF-κB is an important transcription factor that mediates the inflammatory response, while NF-κB/IκB and Nrf2/Keap1 are two important switches for the regulation of oxidative stress (Sies et al., 2017). Therefore, the anti-inflammatory effect of Sal B may be mediated by its regulation of Nrf2/Keap1. Several studies have shown that Sal B can inhibit the expressions of TNF-α, IL-1β, IL-6 and other pro-inflammatory factors in a variety of diseases to exert a good anti-inflammatory effect, and its mechanisms mainly involve the TNF-α/NF-κB signaling pathway and TLR pathway (Zeng et al., 2015; Zhang et al., 2015; Xu et al., 2017) (Table 1; Figure 5). Most studies involve the protective effect of Sal B in neuroinflammation, and middle cerebral artery occlusion (MCAO) is obviously a commonly used model for rat brain I/R injury, which may be more suitable for the pathological state of human cerebral ischemia.

**TABLE 1:** Anti-inflammatory effects of Sal B.

Animal models/cell types	Stimuli	Concentration of Sal B	Mechanisms	References
***In vivo***				
Depressive (mouse)	CMS	20 mg/kg (i.p.)	TNF-α, IL-1β, IFN-γ, iNOS, IL-6↓	(Zhang et al., 2017a)
			Arg-1, TGF-β, IL-4, Ym-1↑	
Depressive (rat)	CMS	20, 40 mg/kg (i.p.)	MDA,IL-6, IL-1β, TNF-α, NLPR3, ASC, cleaved caspase-1↓	(Huang et al., 2019)
			CAT, SOD, GPx↑	
Cerebral I/R injury (rat)	MCAO	30 mg/kg (i.g.)	IL-1β, IL-6, TNF-α↓	(Wang et al., 2016)
			TLR4 signaling pathway (TLR4, MyD88, TRAF6, NF-kB)↓	
Cerebral I/R injury (rat)	MCAO	3, 6, 12 mg/kg (i.p.)	ICAM-1IL-1βIL-6IL-8, MCP-1↓	(Xu et al., 2017)
			NF-κB signaling pathway↓	
Cerebral I/R injury (rat)	MCAO	25 mg/kg (i.p.) (twice)	TNF-α, IL-1β↓	(Lv et al., 2015)
Cerebral I/R injury (mouse)	MCAO	10, 20, 40, 60 mg/kg (*)	ROS,IL-1β, IL-6, TNF-α↓	(Fan et al., 2018)
Spontaneously hypertensive rats	—	80 mg/kg (*)	TNF-α, IL-1β, IL-6, IL-18, MDA↓	(Wang and Hu, 2018)
			SOD, CAT and GSH↑	
Pulmonary inflammation (mouse)	Bleomycin	10 mg/kg (i.p.)	IL-1β, IL-6, COX-2↓	(Liu et al., 2018)
Atherosclerosis (ApoE^−/−^ mouse)	High fat diet	30 mg/kg (i.p.)	IL-6IL-1βTNF-α↓	(Yang et al., 2020)
Cholestatic liver injury (rat)	ANIT	15, 30 mg/kg (i.p.)	IL-1βIL-6TGF-βTNF-α, COX-2↓	(Li et al., 2019)
			NF-κB, p38-MAPK and JNK signaling pathway↓	
Chronic ALD (rat)	Ethanol	15, 30 mg/kg (i.g.)	TNF-α, IL-6, CRP, ChREBP↓	(Zhang et al., 2017b)
NAFLD (rat)	High fat diet	15, 30 mg/kg (i.g.)	HMGB1, TNF-α, IL-8, pro-IL-1β, IL-1β, TLR4, NF-κB↓	(Zeng et al., 2015)
NASH (rat)	High fat diet	20 mg/kg (i.g.)	NF-κB, MDA↓	(Wang et al., 2015)
			SOD↑	
Renal I/R injury (rat)	Nephrectomy	20, 40 mg/kg (i.p.)	NF-κB p65, IL-1β, IL-6, TNF-α, MDA, MPO↓	(Ma et al., 2017)
			SOD, GSH, CAT↑	
			PI3K/Akt signaling pathway↑	
IBD (rat)	TNBS	20, 80 mg/kg (i.g.)	TNF-α, IL-1β, IL-6, MPO, NOX4, iNOS, ROS, MDA↓	(Xiong et al., 2016)
			GSH SOD↑	
Rheumatoid arthritis (rat)	CIA	20, 40 mg/kg (i.p.)	IL-1β, IL-6, IL-17, TNF-α↓	(Xia et al., 2018)
			SODCAT, GSH↑	
			NF-κB signaling pathway↓	
Thromboangiitis obliterans (rat)	Injection sodium laurate into the femoral artery	10, 20, 40 mg/kg (tail i.v.)	TNF-α, iNOS↓	(Zhang et al., 2020)
Asthmatic (mouse)	Ovalbumin	50 mg/kg (i.g.)	IL-13, IL-4, IL-5, MUC5AC, MUC5B↓	(Guan et al., 2018)
			Erk1/2 and P38 signaling pathways↓	
***In vitro***				
Primary microglia cells	LPS	40 *µ*M	INF-γ, TNF-α, IL-6, iNOS, IL-1β↓	(Zhang et al., 2017a)
			IL-4, IL -10, Arg-1, IL-13↑	
Platelet	Collagen I	25, 50, 100 *µ*M	HSP70↑	(Ma et al., 2011)
			Ca^2+^, ROS↓	
Primary cortical neurons cells and PC12 cells	OGD/R	200, 400,800 ng/ml	IL-1β, IL-6, TNF-α↓	(Wang et al., 2016)
			TLR4/MyD88 signaling pathway (TLR4, MyD88, TRAF6, NFkB)↓	
Human umbilical vein endothelial cell line EA.hy926	H_2_O_2_	50 *μ*g/ml	IL-1β, IL-6, COX-2↓	(Liu et al., 2018)
			MAPK and NF-κB signaling pathways↓	
Human acute monocytic leukemia cell line THP-1	LPS	20 *μ*g/ml	IL-1β, TNF-α↓	(Jiang et al., 2020)
Human umbilical vein endothelial cell line EA.hy926	Co-culture with ADP-activated platelets	300 and 600 *μ*g/ml	ICAM-1IL-1βIL-6IL-8MCP-1↓	(Xu et al., 2015)
Human aortic smooth muscle cells	LPS	10 *μ*M	COX-2, ICAM-1↓	(Chen et al., 2006)
			Inhibit ERK and JNK signaling pathways	
HepG2	Palmitic acid	8 *μ*M	HMGB1, TNF-α, IL-8, pro-IL-1β, IL-1β, TLR4, NF-κB↓	(Zeng et al., 2015)
Human primary chondrocytes	IL-1β	25, 50, 100 *μ*M	NO, iNOS, COX2↓	(Lou et al., 2017)
			NF-κB signaling pathway↓	
Human monocyte-derived dendritic cells	Ox-LDL	10, 50, 100 *μ*M	IL-12IL-10TNF-α↓	(Sun et al., 2011)
			TLR4/p38-MAPK signaling pathway↓	

*, Unknown; CMS, chronic mild stress; MCAO, middle cerebral artery occlusion; OGD/R, oxygen-glucose deprivation and reoxygenation; TNBS, 2, 4, 6-trinitrobenzene sulfonic acid; IBD, Inflammatory bowel disease; CIA, collagen-induced rheumatoid arthritis; ADP, adenosine Diphosphate; i.p., intraperitoneally; i.g., intragastrically; i.v., intravenous injection.

#### Tumor Necrosis Factor-α/Nuclear Factor κB Signaling Pathway

NF-κB can rapidly activate the expression of genes involved in the inflammatory response, upregulate the expression of pro-inflammatory factors such as TNF-α, and further form a positive feedback loop to promote the inflammatory response (Hayden and Ghosh, 2014). In a variety of animal model studies of brain injury or nerve injury, Sal B showed anti-inflammatory activity to significantly reduce the expression of pro-inflammatory factors IL-1β and TNF-α (Wang et al., 2016; Fan et al., 2018), and increase the expression of anti-inflammatory factors IL-10 (Chen et al., 2011; Zhang et al., 2016). Studies *in vitro* have shown that Sal B play the role of preventing neuroinflammation by inhibiting the TNF-α/NF-κB signaling pathway (Wang et al., 2010), inhibiting the excessive activation of astrocytes and microglia, and promoting the conversion of activated microglia phenotype from M1 to M2 (Zhang et al., 2017; Yin et al., 2018).

In addition, damaged tissues can promote platelet aggregation through adhesion molecules, and activated platelets are initiators and/or enhancers of the inflammatory response (Nieswandt et al., 2011). Sal B can significantly inhibit the adhesion and aggregation of platelets in the rabbit model of diffuse intravascular coagulation induced by lipopolysaccharide (LPS) (Wu et al., 2014). Further studies on endothelial cells have shown that Sal B can inhibit the expression of platelet P-selectin (Stumpf et al., 2013), inhibit the expression and release of adhesion molecules and chemokines, and attenuate platelet-induced endothelial cell inflammation by inhibiting NF-κB activation (Xu et al., 2015). These results suggesting that the anti-inflammatory effects of Sal B in cardiovascular system disease may be partly related to its anti-coagulant effects, and inhibiting TNF-α/NF-κB signaling is one of the mechanisms of anti-coagulant effect of Sal B. In addition to regulating Nrf2, ROS can also regulate the activation and translocation of NF-κB into the nucleus to activate the downstream target genes (Ma, 2013). Oxidative stress may be involved in the regulation of the TNF-α/NF-κB signaling pathway, suggesting that the anti-inflammatory effect of Sal B is inseparably related to its antioxidant effect.

#### Toll-Like Receptor Pathway

TLR4 is an innate immune receptor involved in the inflammatory response. Damage-related molecules patterns and LPS can trigger TLR4 signaling and activate myeloid differentiation factor MyD88, thereby increasing the transcriptional activity of NF-κB and triggering the inflammatory response (Rahimifard et al., 2017). In the MCAO-induced rat brain I/R injury model, Sal B can inhibit TLR4-mediated NF-κB inflammatory pathway through MyD88-dependent pathway (Wang et al., 2016). It can also inhibit the TLR4-mediated p38-mitogen-activated protein kinase (MAPK) signaling pathway to inhibit the maturation of monocyte-derived dendritic cells *in vitro* (Sun et al., 2011). These results suggest that Sal B can exert anti-inflammatory effects by inhibiting TLR4-mediated NF-κB and MAPK signal pathways. In addition, in the study of MAFLD (NAFLD) characterized by hepatic steatosis and inflammation, Sal B can also inhibit the migration and release of high mobility group protein 1 (HMGB1) by upregulating SIRT1 expression (Zeng et al., 2015). HMGB1 can aggravate inflammatory damage by enhancing the TLR4/MyD88-dependent pathway (Li et al., 2011), and SIRT1 can directly inhibit TNF-α/NF-κB signaling (Cui et al., 2016), which further demonstrate the correlation between the antioxidant and anti-inflammatory effects of Sal B.

### Antioxidant and Anti-Apoptotic Effects of Salvianolic Acid B

The damage of ROS-induced lipid peroxidation to biological membranes is an important mechanism leading to cell apoptosis. Lipid peroxidation can damage phospholipids directly, and can also induce apoptosis as a cell death signal (Su et al., 2019). In addition to inhibiting MDA and ROS, Sal B can also regulate multiple signaling pathways to up-regulate the expression of anti-apoptotic protein B-cell lymphoma 2 (Bcl-2) and down-regulate the expression of pro-apoptotic protein Bax in a variety of disease models. These results suggest that the anti-oxidation and anti-apoptotic effects of Sal B have a certain correlation (Table 2). So far, most studies have focused on two acute diseases, liver injury and AMI, and arterial ligation surgery is undoubtedly the most commonly used model for cardiovascular disease research.

**TABLE 2:** Anti-apoptosis effects of Sal B.

Animal models/cell types	Stimuli	Concentration of Sal B	Mechanisms	References
***In vivo***				
Hepatic injury (mouse)	d-galactosamine and LPS	10 mg/kg (i.g.)	—	(Yan et al., 2010)
AMI (rat)	Ligation of the left anterior descending coronary artery	10 mg/kg (i.v.)	PARP-1, cleaved-PARP-1↓	(Xu et al., 2011)
Vascular dementia (rat)	Permanent bilateral common carotid artery occlusion	20 mg/kg (i.g.)	IGF-1/Akt Pathway↑	(Xiaowei Ma et al., 2017)
AMI (rat)	Ligation of the left anterior descending coronary artery	1.5, 3, 6, 12, 24 mg/kg (i.v.)	LDH, CK, MDA, Bax, cleaved Caspase-9, cleaved PARP↓	(Lin et al., 2016)
		30, 60, 120, 240, 480 mg/kg (i.g.)	Bcl-2, SOD, LC3-II, Beclin1, VEGF↑	
Cardiac injury (rat)	Doxorubicin	0.25, 0.5, 1 mg/kg (i.v.)	—	(Chen et al., 2017)
***In vitro***				
Human hepatocyte cell line HL-7702	Actinomycin D and TNF-α	1 *μ*M	TNF-a, TNF-R1, cytochrome C, Caspase-3↓	(Yan et al., 2010)
			Bcl-2↑	
Rat H9c2 cells	Hypoxia	1, 10 *μ*M	-	(Xu et al., 2011)
Rat H9c2 cells	ATO	0.1, 1, 10 *μ*M	ROS, Caspase-3, Bax↓	(Wang et al., 2013)
			Bcl-2, Bcl-xl↑	
			PI3K/Akt signal pathway↑	
Rat BMSCs	H_2_O_2_	10 *μ*M	Caspase-3↓	(Lu et al., 2010)
			Bcl-2↑	
			ROS/MEK/ERK signal pathway↓	
Mouse embryonic hepatocyte cell line BNL CL.2	H_2_O_2_	10 *μ*M	PCC, CatB, CatD↓	(Yan et al., 2017)
			LAMP1↑	
HUVECs	H_2_O_2_	10 nM, 100 nM, 1 *μ*M	GRP78 and GRP94, PERK, eIF2a, ATF4, ATF6↑	(Wu et al., 2009)
Primary rat cardiac ventricular myocytes	Doxorubicin	20 *μ*g/ml	Ca^2+^, Bax, GRP78, CHOP, TRPC3, TRPC6↓	(Chen et al., 2017)
			Bcl-2 ↑	
Isolated rabbit'|’s thoracic aortic rings	Phenylephrine	1, 2, 4 mg/ml	Ca^2+^↓	(Shou et al., 2012)
			NO-sGC-cGMP signal pathway↑	

ATO, Arsenic trioxide; AMI, acute myocardial infarction; BMSCs, bone marrow stem cells; PCC, protein carbonyl content; HUVECs, human umbilical vein endothelial cells; PERK, pancreatic ER kinase (PKR)-like ER kinase; ATF, activating transcription factor; i.g., intragastrically; i.v., intravenous injection.

#### Classical Apoptotic Pathway

Classical cell apoptotic pathways include the death receptor pathway and the mitochondrial pathway. Sal B can inhibit the expression of TNF-α and TNF-R1 and inhibit the nuclear translocation of NF-κB (Yan et al., 2010; Wang et al., 2012), so its mechanism of inhibiting cell apoptosis by regulating the death receptor pathway is closely related to its antioxidant and anti-inflammatory effects. Excessive ROS can directly damage the structure and function of mitochondria and induce cell apoptosis. Numerous studies in rat models of cardiovascular diseases have shown that Sal B can improve the integrity of mitochondria and nuclei by inhibiting the PARP-1 pathway, and activating the insulin-like growth factor-1/Akt signaling pathway to exert anti-apoptotic effects (Xu et al., 2011; Lin et al., 2016; Ma et al., 2017). At the same time, Sal B can also activate PI3K/Akt pathway and inhibit the MEK/ERK signaling pathway to promote the expressions of Bcl-2 and B-cell lymphoma-extra large, and inhibit the expression of Bax in in vitro studies (Lu et al., 2010; Wang et al., 2013a). Indicating that Sal B has an effect on mitochondrial-dependent apoptosis. ROS can also damage mitochondria and induce apoptosis by affecting lysosomal membrane permeability to release of hydrolase (Gao C. et al., 2014). In the H_2_O_2_-induced apoptosis of BNL CL.2, a mouse liver cell line, Sal B can stabilize lysosomal membranes by increasing the expression of lysosomal-associated membrane protein 1 and antagonizing cathepsin B/D leakage into the cytoplasm (Yan et al., 2017). These results indicate that both the death receptor pathway and the mitochondrial pathway are related to excess ROS overdose, and the inhibitory effect of Sal B on these two classical apoptotic pathways may be a continuation of its antioxidant effect.

#### Non-classical Apoptotic Pathway

Endoplasmic reticulum (ER) stress is a non-classical cell apoptotic pathway, and glucose regulatory protein 78 (GRP78) is a central regulator of ER stress. In the endothelial cell injury model induced by oxidative stress, Sal B inhibits apoptosis by promoting the expression of GRP78 to inhibit ER stress (Wu et al., 2009), which further suggested the correlation between antioxidant and anti-apoptotic effects of Sal B. In addition, ion channels are one of the main mechanisms that transmit external signals across the cell membrane to the inside. ROS can directly post-translationally modify channel proteins or change the activity of other signal transduction factors, which in turn leads to changes in activity of channel proteins or expression of channel genes. Sal B can inhibit extracellular Ca^2+^ influx by inhibiting classical transient receptor potential channels 3 (TRPC3) and TRPC6, and inhibit intracellular Ca^2+^ release in a ryanodine receptor-dependent manner (Shou et al., 2012; Chen et al., 2017). These studies have shown that Sal B can alleviate Ca^2+^ overload and inhibit apoptosi*s* by regulating the intracellular Ca^2+^ concentration, and its mechanism may be partly depend on the NO-sGC-cGMP signaling pathway. In addition, Sal B has shown an inhibitory effect on other members of the TRP superfamily, such as TRPM6 and TRPM7 (Yang et al., 2018). Previous studies have confirmed that ROS can regulate the TRP family and thus regulate the Ca^2+^ signaling cascade (Song et al., 2011), and Ca^2+^ signal-mediated ER stress is a non-classical apoptotic pathway. Sal B inhibits ER stress-induced cell apoptosis by regulating Ca^2+^ channels, which may be related to its clearance and inhibition of ROS production.

### Antioxidant Effect and Autophagy Regulation of Salvianolic Acid B

Autophagy dysfunction is closely related to inflammatory diseases, and the recovery of autophagy can improve many chronic inflammatory diseases (Cadwell, 2016). This may be due to the active participation of autophagy in the elimination of inflammatory bodies and pro-inflammatory cytokines, and its ability to regulate the balance of anti-inflammatory and inflammatory responses (Zhong et al., 2016). Microtubule-associated proteins light chain 3 (LC3) reflects the activity of autophagy. Sal B has a regulatory effect on LC3 in some studies, but the results are conflicting (Table 3). In the LPS-induced depression rat model, Sal B can up-regulate the expression of LC3 to promote autophagy and nod-like receptor family pyrin domain containing three clearance in the hippocampus (Jiang et al., 2017). To the opposite, Sal B can inhibit the autophagy in the rat Schwann cells treated with high glucose by down-regulating the JNK signaling pathway (Wang et al., 2019), and inhibit the autophagy of the starvation model of cardiomyocytes by activating PI3K/Akt signaling *in vitro* (Han et al., 2011; Dan Li et al., 2016). However, it is worth noting that the pros and cons of autophagy in pathological conditions are not very clear (White, 2012). Mitochondria are the main source of ROS, and ROS-activated mitochondrial autophagy can relieve oxidative stress (Scherz-Shouval and Elazar, 2011b). The different regulatory effects of Sal B on autophagy may be related to the degree of cell damage and the form of autophagy. It may play a protective role in the early stage of autophagy and promote the expressions of autophagy and inflammatory factors in the late stage, which should be further confirmed.

**TABLE 3:** Autophagy regulation effect of Sal B.

Animal models/cell types	Stimuli	Concentration of Sal B	Mechanisms	References
***In vivo***				
Depression (rat)	LPS	20 mg/kg (i.g.)	IL-1β, IL-6, NLRP3, ASC, caspase-1 P20, IL-1β↓	(Jiang et al., 2017)
			LC3, LC3-II/I ratio, Beclin-1↑	
***In vitro***				
RSC96 cells	High glucose	0.1, 1, 10 *μ*M	PARP, cleaved caspase 3, cleaved caspase 9, LC3A/B, Beclin1↓	(Wang et al., 2019)
			Bcl-2↑	
			JNK signaling pathway↓	
Primary rat ventricular myocytes	Starvation	50 *μ*M	LC3-II, Caspase-8↓	(Han et al., 2011)
			Cellular ATP content↑	
Primary mouse myocardial cells	OGD	25, 50, 100 *μ*M	LC3-II, beclin-1, LDH leakage↓	(Dan Li et al., 2016)
			miR-30a↑	
			PI3K/Akt signaling pathway↑	

OGD, oxygen-glucose deprivation; i.g., intragastrically.

### Antioxidant and Anti-Fibrotic Effects of Salvianolic Acid B

Myofibroblasts (MFBs) proliferation is the core event of fibrosis. In the liver, activated hepatic stellate cells (HSCs) are the main source of MFBs. ROS is closely related to HSCs activation, and activated HSCs can also release ROS, further aggravating liver damage and fibrosis. The current anti-fibrosis research of Sal B mainly focuses on the liver. In addition to the regulation of HSCs and liver sinusoid endothelial cells (LSECs), the improvement of serum ALT and AST of Sal B in a variety of animal models of hepatic fibrosis also shows the improvement of oxidative damage of hepatocytes of Sal B. It has been shown that Sal B have an good antioxidant effect in the hepatic fibrosis models induced by chemical drugs such as TAA, DMN and CCl_4_ (Table 4).

**TABLE 4:** Anti-fibrotic effect of Sal B.

Animal models/cell types	Stimuli	Concentration of Sal B	Mechanisms	References
***In vivo***				
Hepatic fibrosis (rat)	TAA	50 mg/kg (i.g.)	ALT, AST, fibrosis score, HO-1, iNOS, TNF-α, IL-6, and IL-1β, MDA, α-SMA, gp91^phox^, p47^phox^↓	(Tsai et al., 2010)
			GSH↑	
Hepatic fibrosis (rat)	DMN	10 mg/kg (i.g.)	α-SMA, TGF-β1, TβR-I, and TβR-II↓	(Tao et al., 2013)
Hepatic fibrosis (rat)	DMN	10 mg/kg (i.g.)	Col-I, α-SMA, AT1R, ERK/pERK	(Li et al., 2012)
Chronic pancreatitis (rat)	DBTC	10 mg/kg (i.g.)	AST, ALT, BUN, Cr, MDA, α-SMA, Col-I, TGF-β, p-Smad2/3↓	(Xu et al., 2016)
			SOD, Smad7↑	
Systemic sclerosis (mouse)	Bleomycin	10 mg/kg (i.p.)	Col1a1, Col1a2, Col3a1, Ctgf, PAI-1↓	(Liu et al., 2019)
Hepatic fibrosis (mouse)	CCl_4_	100 mg/kg (i.g.)	α-SMA, Col-I, desmin, vimentin, Smo, Gli2 and, DNMT1↓	(Yu et al., 2015)
			E-cadherin, PTCH1↑	
Liver cirrhosis (rat)	DMN	12.5 mg/kg (i.g.)	ALT, AST, bilirubin, Hyp	(Xu et al., 2012)
***In vitro***				
HSC-T6 cells	PDGF	200, 300, 400 *μ*M	HO-1, Nuclear factor E2-related factor 2, p-JNK, p-p38, Trx, p-PKC-δ, p-PKD↓	(Tsai et al., 2011)
HSC-T6 cells	PDGF	200 *μ*M	ROS, α-SMA, gp91^phox^, p47^phox^↓	(Tsai et al., 2010)
Primary rat HSCs	TGF-β1	1, 10 µM	α-SMA, TβR-I, T[1]II, TβRI, Smad3, p-Smad3↓	(Tao et al., 2013)
Primary rat HSCs	TGF-β1	10 *μ*M	α-SMA, Col-I, p-MEK, p-ERK, p-Raf, p-p38, MEF2C, MEF2A, p-MKK3/6↓	(Lv et al., 2010)
			ERK and p38 MAPK signaling pathways↓	
Primary mouse HSCs	-	10 *μ*M	α-SMA, Col-I, desmin, vimentin, Smo, Gli2, DNMT1↓	(Yu et al., 2015)
			Caspase3, E-cadherin, PTCH1↑	
MRC5 cells	TGF-β1	1, 10 *μ*M	Col-I, TGF-β1, α-SMA↓	(Zhang et al., 2014)
Primary rat PSCs	-	100 *μ*M	α-SMA, Col-I, Vimentin,TGF-β, p-Smad2/3↓	(Xu et al., 2016)
			E-cadherin, Smad7↑	
Human skin fibroblasts	TGF-β1	50 *μ*g/ml	COL1 A1, COL1 A2, COL3 A1, CTGF, FN1, PAI-1, α-SMA, p-Smad3, p-ERK↓	(Liu et al., 2019)
HSC-T6 cells, primary rat HSC cells	Ang II	1, 10 nM	α-SMA, Col-I, TGF-β, ERK, pERK, c-Jun, p-c-Jun	(Li et al., 2012)
Primary rat CFBs	Ang II	12.5, 25, 50 *μ*M	Col-I,α-SMA, FN, CTGF, p-IκBα, IκBα, p-p65, p65↓	(Wang et al., 2018)
			NF-κB signaling Pathway↓	
Primary rat HSCs	ET-1	100 *μ*M	p-MLC2, Ca^2+^, RhoA activity, ROCK II↓	(Xu et al., 2012)
Primary rat LSECs	LPS	200 *μ*M	MCP-1↓	(Chen et al., 2013)

TAA, thioacetamide; DMN, dimethylnitrosamine; DBTC, dibutyltin dichloride; Hyp, hydroxyproline; PDGF, platelet-derived growth factor; PSCs, pancreatic stellate cells; Trx, thioredoxin; CFBs, cardiac fibroblasts; ET-1, endothelin-1; LSECs, liver sinusoidal endothelial cells; i.p., intraperitoneally; i.g., intragastrically; i.v., intravenous injection.

**TABLE 5:** The regulation effect of Sal B on microcirculation.

Animal models/cell types	Stimuli	Concentration of Sal B	Mechanisms	References
***Vivo***				
spinal Cord injury (rat)	Dural compression	1, 10, 50 mg/kg (i.v.)	TNF-α, NF-κB↓	(Fan et al., 2013)
			ZO-1, occludin, HO-1↑	
pulmonary Microcirculation disturbance (rat)	LPS	5 mg/kg (i.g.)	E-selectin, ICAM-1, IL-8, TNF-α, MPO, MMP-2, MMP-9↓	(Lin et al., 2013)
			AQP-1, AQP-5↑	
***Vitro***				
Primary rat brain microvascular endothelial cells	Glucose	10, 20, 100 *μ*M	ROS, HIF-1α, VEGF↓	(Yang et al., 2016)
			ZO-1, occludin, miR-200b↑	
HUVECs	LPS	10 *μ*M	Caveolin-1, p-VE-cadherin↓	(Pan et al., 2015)
			F-actin, ZO-1↑	

i.g., intragastrically; i.v., intravenous injection.

**TABLE 6:** Regulation of Sal B on stem cells.

Animal models/cell types	Stimuli	Concentration of Sal B	Mechanisms	References
Mouse ESCs	LIF	0.001, 0.01, 0.1, 1 nM	Oct4, Sox2, AP, Nanog, SSEA1, IL-5, IL-11, EGF, CNTF, EPO, IL-6↑	(Chia Hui Liu et al., 2014)
			Jak2-Stat3 and EGFR-ERK1/2 signaling pathways↑	
Human ESC line H9	Activin A	0.5, 1, 10, and 20 *μ*M	ALB, Ki67, β-catenin, LEF1, TCF3, cyclin D1, c-myc, Wnt1, Wnt2, Wnt3, Wnt6, Wnt7a, MMP7↑	(Chen J. et al., 2018)
			Notch1, Notch3, Jagged1, Hes1, Hes5, CCL5, CK7↓	
			Wnt Pathway↑	
			Notch Pathway↓	
Mouse iPSCs	Retinoic acid	5, 50, 100 *μ*M	Nestin, Cyclin-D, MAP2↑	(Shu et al., 2018)
			pβ-catenin↓	
			PI3K/AKT/GSK3β↑	

Oct4, Octamer-binding transcription factor 4; Sox2, sex-determining region Y box 2; AP, alkaline phosphatase; SSEA1, stage-specific embryonic antigen one; iPSCs, induced pluripotent stem cells.

**TABLE 7:** Anti-cancer effect of Sal B.

Animal models/cell types	Stimuli	Concentration of Sal B	Mechanisms	References
***In vivo***				
Breast cancer (nude mouse)	MDA-MB-231 cells	80 mg/kg (i.p.) (three times per week)	PCNA, Bcl-xL, survivin↓	(Sha et al., 2018)
Colorectal cancer (nude mouse)	HCT116 cells	80 mg/kg (i.p.)	LC3-II↑	(Jing et al., 2016)
			AKT/mTOR signaling pathway↓	
HNSCC (Nude mouse)	JHU-013 cells	40, 80 mg/kg (i.p.)	PCNA, COX-2↓	(Zhao et al., 2010)
Cardiotoxicity (mouse)	ATO	2 mg/kg (tail vein injection)	CK, LDH, GOT, CAT, Bax↓	(Wang et al., 2013b)
			SOD, GSH-PX, Bcl-2, p-Akt↑	
Squamous cell carcinoma (hamster)	DMBA	10 mg/kg (i.g.)	Glutaminolysis, glycolysis, inflammation, tumor angiogenesis↓	(Wei et al., 2012)
			Cholesterol and myo-inositol metabolism↑	
***In vitro***				
Osteosarcoma cell line MG63	—	1, 10, 50, 100 µM	Cleaved Caspase-3, p-p38, p-p53, ROS↑	(Zeng et al., 2018)
Human breast cancer cell lines MCF-7 and MDA-MB-231	—	50, 100, 150, 200 *μ*M	Cyclin B1, Bcl-xL, survivin, GCS, GM3 enzymes↓	(Sha et al., 2018)
			ceramide↑	
Human colon cancer cell lines HCT116 and HT29	BafA1	25, 50, 100, 200,400 *μ*M	LC3-II/I ratio, cleaved PARP, cleaved-Caspase-9, cleaved-Caspase-3↑	(Jing et al., 2016)
			AKT/mTOR signaling pathway↓	
Hepatocellular carcinoma cell lines SK-Hep-1 and Bel-7404	—	100, 200 *μ*M	Cleaved PARP, cleaved Caspase-3, cleaved Caspase-9, cytochrome c, LC-3, p62, Beclin-1↑	(Gong et al., 2016)
			p-AKT, p-mTOR, p-4EBP1, p-P70S6K↓	
			AKT/mTOR signaling pathway↓	
HNSCC Cell lines JHU-013, and JHU-022	-—	50, 100 *μ*M	COX-2, PGE2, Bcl-2, EGFR↓	(Zhao et al., 2010)
			p53↑	
HepG2, HeLa cells	ATO	10 *μ*M	Procaspase-3↓	(Wang et al., 2013b)
			Cleaved PARP↑	
Human glioma U87 and U373 cells	Radiation, temozolomide	0.5 *μ*M	`^2+^ buffering capacity↓	(Chen W. et al., 2018)
			Fis-1↑	
OSCC Cell lines CAL27 and SCC4	—	50, 100, 200 *μ*g/ml	CAL27	(Yang et al., 2011)
			Tenascin C, Osteoponti, HIF-1α, TGFb1, COX-2, HGF, Scya2, IL-10, TGFbR2, Mmp2↓	
			THBS2↑	
			SCC4	
			HIF-1α, Mmp9, TGF b3, VEGF, VEGF-C, TNFa↓	
			THBS2, Timp1↑	
Human GC cell line BGC-823	—	200 *μ*M	citH3, MPO↓	(Tao et al., 2018)

DMBA, 7,12-dimethylbenz(a)anthracene; BafA1, bafilomycin A1; HNSCC, head and neck squamous cell carcinoma; OSCC, oral squamous cell carcinoma; i.p., intraperitoneally; i.g., intragastrically.

Sal B has a significant inhibitory effect on ROS generation in HSCs model induced by platelet derived growth factor *in vitro* (Tsai et al., 2010; 2011), suggesting that the inhibition effect of HSCs activation of Sal B is related to its antioxidant activity. Further studies have found that Sal B can inhibit the TGF-β1/Smads signaling pathway in the activation and proliferation of HSCs and pancreatic stellate cells treated with TGF-β1 (Tao et al., 2013; Xu et al., 2016), and also inhibit the MAPK pathway in the proliferation of skin fibroblasts (Liu et al., 2019). The MAPK pathway includes three types: extracellular regulated protein kinases (ERK) pathway, JNK pathway, and p38 MAPK pathway (Kumar et al., 2003). Sal B can inhibit both the ERK and p38 MAPK pathways (Lv et al., 2010; Guan et al., 2018). Furthermore, angiotensin II (Ang II) can regulate the activation of HSCs or cardiac fibroblasts to MFB through the Ang II receptor type 1 (AT1R) or NF-κB pathway, increase the secretion of cytokines including TGF-β1 and connective tissue growth factor (CTGF), and increase the expression of collagen (Douillette et al., 2006; Xu Li et al., 2007; Tian et al., 2015). Sal B not only inhibits the expression of TGF-β1 stimulated by Ang II and thereby inhibits HSCs activation by reducing AT1R expression (Shu Li et al., 2012), but also reduces the proliferation and migration of MFB differentiated from cardiac fibroblasts induced by Ang II through inhibiting the NF-κB pathway (Wang et al., 2018). In addition, Sal B also has inhibitory effects on the epithelial-mesenchymal transition (Tang et al., 2014; Yu et al., 2015). After HSCs activation, it can contract and compress the liver sinus, and then change the liver microcirculation. The RhoA/ROCK pathway is considered to be the main signaling pathway that controls HSCs contraction (Melton et al., 2006). Sal B can inhibit the contractility of HSCs by inhibiting the RhoA/ROCK II pathway (Hong Xu et al., 2012), and inhibit LSECs dedifferentiation induced by LPS through inhibiting the expression of monocyte chemotactic protein 1, to inhibit the migration of HSCs (Chen H. et al., 2013). These results indicate that Sal B not only directly inhibits the proliferation of MFB but also inhibits the proliferation and migration of HSCs-activated MFB by inhibiting LSECs dedifferentiation.

The studies *in vivo* on rat models of hepatic fibrosis and mouse models of systemic sclerosis have further demonstrated that the regulation of Sal B on TGF-β1/Smads, MAPK, Ang-II and RhoA/ROCK signaling pathways (Xu et al., 2012; Li et al., 2012; Tao et al., 2013; Liu et al., 2019). ROS can activate TGF-β1 signaling by not only oxidizing latency-associated protein, but also activating matrix metalloproteinases (MMPs) such as MMP-2 and MMP-9 (Liu and Desai, 2015). It can also up-regulate the expression of TGF-β gene in various types of cells (Liu and Desai, 2015), and mediate ERK and p38 phosphorylation to regulate the MAPK pathway (Fang et al., 2018; Zhou et al., 2020). Therefore, the inhibition by TGF-β1/Smads and MAPK signaling pathways of Sal B may be indirect effects.

### Antioxidant and Regulating Microcirculation and Barrier Function

Vascular endothelial growth factor (VEGF) can downregulate the endothelial transmembrane tight junction proteins claudin-5 and occludin (OCLN) (Argaw et al., 2009), and upregulate the cell membrane caveolin proteins caveolin-1 and caveolin-2 (Ba et al., 2014), changing endothelial cell permeability through paracellular and transcellular pathways. The MMPs family, including MMP-2 and MMP-9, regulates the integrity of the extracellular matrix and disrupts tight junctions between cells (Rosenberg, 2009; Bauer et al., 2010). In high glucose-induced rat brain microvascular endothelial cells (RBMECs) model, Sal B can inhibit ROS/hypoxia inducible factor 1 alpha/VEGF signal, reduce the permeability of RBMECs, to improve blood-brain barrier dysfunction (Yang et al., 2016). The study of rat spinal cord injury model shows that Sal B can repair the blood spinal cord barrier through decreasing the expressions of HO-1, TNF-α and NF-κB, and increasing the expressions of OCLN and ZO-1 (Fan et al., 2013), which indicates that the regulation of endothelial cell permeability by Sal B is closely related to its antioxidant effect. At the same time, Sal B can reduce the expression of IL-8, TNF-α, MPO, MMP-2, and MMP-9 in rats with LPS-induced pulmonary microcirculation disorder (Lin et al., 2013), suggesting that Sal B can regulate the barrier function between cells to improve cell permeability. These results suggest that to a certain extent, the improvement of blood spinal cord barrier, blood-brain barrier and intestinal barrier function of Sal B is the result of antioxidation. In addition, ROS can activate the tyrosine kinase Src through a variety of mechanisms (MacKay and Knock, 2015), and Src can regulate endothelial permeability through paracellular and transcellular pathways (Hu et al., 2008). In addition to inhibiting the production of ROS through antioxidant effects, Sal B can also bind to the SH2 domain of Src to inhibit the phosphorylation of Src (Sperl et al., 2009; Pan et al., 2015), suggesting that Src may be a direct target of Sal B to regulate cell permeability.

### Antioxidant and Promote Stem Cell Proliferation and Differentiation

Stem cells are highly proliferative and can differentiate into one or more functional cells. ROS plays a vital role in maintaining the stemness and differentiation of stem cells (Chaudhari et al., 2014). Pluripotent stem cells include embryonic stem cells (ESCs) and induced pluripotent stem cells (iPSCs). Previous studies have shown that Sal B can promote the proliferation of ESCs through activating Jak2/Stat3 and EGFR-ERK signaling pathways (Liu et al., 2014), and induce differentiation into hepatocytes of ESCs by activating Wnt pathway and inhibiting Notch pathway *in vitro* (Chen J. et al., 2018). Research on the differentiation into neural stem cells of iPSCs shows that Sal B can also up-regulate the PI3K/AKT/GSK3β/β-catenin pathway and enhance the neural differentiation of iPSCs (Shu et al., 2018). The regulation effect of Sal B on multiple signal pathways may be the result of its anti-oxidant intervention (Arany et al., 2006; Dal-Cim et al., 2012; Yang et al., 2013; Zhou et al., 2019). It is worth noting that different differentiated directions of stem cells induced by Sal B may be related to the complex *in vivo* environment. For example, Sal B can promote the differentiation of mesenchymal stem cells (MSCs) into endothelial cells instead of cardiomyocytes *in vivo* by activating Wnt/β-catenin signaling pathway (Gao Q. et al., 2014). Sal B can be used as a potential agent for stem cells therapy, and may need to be used in combination with corresponding differentiation regulators, which needs to be further proven.

## Inhibit Cancer Cells Proliferation and Metastasis *In Vitro*

For cancer cells, Sal B has the opposite effect on regulating cell apoptosis. Previous studies *in vitro* have shown that Sal B can not only inhibit the proliferation and induce apoptosis of osteosarcoma cells by promoting the p38-MAPK pathway to mediate ROS production (Zeng et al., 2018), but also promote the autophagy of colorectal cancer cells and hepatocellular carcinoma cells by inhibiting the Akt/mTOR signaling pathway (Gong et al., 2016; Jing et al., 2016). Since mitochondria are both the main source of ROS and the site of cellular energy metabolism, cell types with abnormal energy metabolism such as cancer cells may have higher levels of ROS to maintain a high proliferation rate. Defects in ceramide production and metabolism contribute to cancer cells survival and resistance to chemotherapy (Morad and Cabot, 2013). Sal B can inhibit the proliferation and induce apoptosis of human breast cancer cell *in vivo* and *in vitro* through inducing ceramide accumulation and ceramide-mediated apoptosis (Sha et al., 2018). Different regulatory effects of Sal B on injured cells and cancer cells may lie in regulating energy metabolism homeostasis and redox homeostasis. The difference in energy metabolism may be one of the key factors affecting the different regulatory effects of Sal B on ROS, but it should be further confirmed.

Sal B can be combined with anti-cancer drugs to enhance their efficacy and reduce their dosage. Both celecoxib and arsenic trioxide have limitations in their long-term application in cancers due to cardiotoxicity. In addition to enhancing the inhibition of cancer cell proliferation and promoting cancer cell apoptosis, previous studies of Sal B *in vivo* and *in vitro* have shown that it can not only reduce the dosage of celecoxib (Zhao et al., 2010), but also improve the myocardial damage of arsenic trioxide (Wang et al., 2013b). Even for radiotherapy, Sal B can make cancer cells more sensitive to radiation through aggravating radiation-induced apoptosis and mitochondrial dysfunction (Chen W. et al., 2018).

In addition, it has been shown that Sal B can increase the expression of angiogenesis-related genes and metabolites in a hamster model of oral squamous cell carcinoma (Wei et al., 2012). The study on oral squamous cell carcinoma cell lines *in vitro* also shows that Sal B can inhibit the expression of angiogenesis-related genes such as HIF-1α, TGF-β1, COX-2, HGF and MMP9 (Yang et al., 2011). These suggest that Sal B also has a certain inhibitory effect on abnormal angiogenesis in cancer. In addition, LPS can trigger platelet activation by upregulating the expression of TLR4, promoting the formation of neutrophil extracellular traps (NETs) (Clark et al., 2007). NETs are a kind of DNA webs derived from neutrophils, which can capture circulating cancer cells and promote cancer metastasis (Cools-Lartigue et al., 2013). In a nude mouse model study of lung metastasis of gastric cancer cells, Sal B can prevent the migration of neutrophils to the metastatic site by reducing plasma neutrophil elastase and fibrinogen levels, and disrupting early NETs formation by blocking myeloperoxidase to prevent hematogenous metastasis of cancer cells (Tao et al., 2018). These results suggest that the anti-inflammatory and anti-oxidant effects of Sal B may be beneficial for suppressing cancer metastasis to a certain extent. However, Sal B has been shown to inhibit cancer cell proliferation, promote cancer cell apoptosis and autophagy *in vitro*, but the evidence *in vivo* is still lacking. Therefore, further studies *in vivo* are needed to demonstrate the effect of Sal B on cancer.

## Low Bioavailability of Salvianolic Acid B

Sal B has strong hydrophilicity, is unstable in aqueous solution, and has a first-pass effect in the liver. It can be quickly absorbed and metabolized into methylated products by hepatocytes through organic anion transporters, and then quickly excreted with bile (Li et al., 2007; Qi et al., 2013). These characteristics affect the bioavailability and biological effects of Sal B. Sal B has shown a wide range of applications *in vivo* and *in vitro*, thus, it is a very important to improve its bioavailability. Chelated magnesium ions contribute to the stability of the molecular structure of Sal B, which has good pharmacological effects but still has low bioavailability (Wu and Wang, 2012). Changing the dosage form or adding antioxidants may improve the stability of Sal B, and different administration methods for different diseases can also improve the bioavailability of Sal B. For example, nasal administration can improve its brain targeting (Zhang et al., 2018). In recent years, microparticle drug delivery systems have developed rapidly. Nanocomplex of Sal B can improve its bioavailability, increase the targeting effect of Sal B on specific tissues and specific cells, and inhibit cancer cells proliferation and promote apoptosis more effectively *in vitro* (Li et al., 2016; Li et al., 2017). Compared with intravenous injection and conventional liposomes, long-circulating liposomes of Sal B prolong the retention time and drug concentration of Sal B in liver, kidney, and brain, showing higher bioavailability (Pi et al., 2015). Sustained release of Sal B using scaffolds has also shown to enhance the osteogenic differentiation and osteogenesis of MSCs during spinal fusion or repair of bone tissue defects (Li et al., 2016; Lin et al., 2019). The current multidisciplinary research provides more possibilities for improving the bioavailability of Sal B, which needs more cooperation in disciplines such as biomaterials and medicinal chemistry.

However, it should be noted that the effect of antioxidants does not increase along with dose. For example, high doses of vitamin C act as oxidants rather than antioxidants (Reczek and Chandel, 2015). A recent study showed that the activation of hepatocyte-specific Nrf2 can promote liver lipid accumulation and glycogen synthesis and induce the expression of growth factors such as TGFα and platelet derived growth factor (He et al., 2020), which suggests that excessive antioxidant may affect liver metabolic function and induce the proliferation of HSCs, while excessive Sal B may further aggravate tissue damage. Therefore, while increasing the bioavailability of Sal B, attention should also be paid to the dose. In the current studies *in vivo* and *in vitro*, the employed doses and concentrations, respectively, of Sal B varies greatly and it is difficult to compare horizontally. The dosage of anti-inflammatory effects of Sal B *in vivo* is mainly 15–80 mg/kg (rats, intragastric administration) and 3–40 mg/kg (rats, intraperitoneal injection). The wide range of dosage shows that its anti-inflammatory and antioxidant effects are its main effect mechanisms. While the same dosage in different models illustrates that the correlation between the antioxidant of Sal B and its extensive pharmacological effects. The dosage of Sal B *in vivo* on anti-cancer study is mainly 80 mg/kg (mice, intraperitoneal injection), which is significantly higher than the dosage in other studies. The concentration and effect of Sal B also showed the same trend *in vitro*. Although this may be due to the abnormal energy metabolism of cancer cells, it also suggests that Sal B may inhibit the generation of ROS to play an anti-oxidant effect at lower doses, and promote ROS production to promote oxidative damage at higher doses.

## Summary

The main role of Sal B is strong antioxidant capacity. Whether by donating hydrogen atoms and stabiling free radicals, or by regulating antioxidant enzymes to inhibit the generation of ROS, Sal B exhibits a powerful regulatory effect on oxidative stress, which may be one of the basis of its anti-inflammatory, anti-apoptotic, anti-fibrotic and other pharmacological effects. Considering that dysregulated redox balance and continuous oxidative damage is the pathological basis of cardiovascular and cerebrovascular diseases, aging, fibrosis and other chronic diseases, Sal B offers interesting prospects to explore in further research on these diseases. Current research on the pharmacological effects of Sal B has not yet elucidated the mechanisms of its extensive pharmacological effects. Most studies have only observed the effects of Sal B on related signaling pathways and oxidative stress indicators, and the regulation of Nrf2/Keap1 pathway may be the core of the pharmacological effects of Sal B, but its specific targets are still unclear. However, it should be noted that under different conditions, the regulatory effect of Sal B on oxidative stress is not the same, which may be related to the cell type and the degree of cell damage. Due to the difference in energy metabolism between cancer cells and normal cells, the role of Sal B in increasing ROS in cancer cells to promote cell apoptosis *in vitro* may be closely related to its abnormal energy metabolism pathway, but the definite effects of Sal B on cancer should be further studied *in vivo*. Therefore, the correlation between the differential energy metabolism of cells of different types or states and oxidative stress may be the key links in the role of Sal B. In addition, the relationship between the dosage of Sal B and its antioxidant effect has not been enough attention. It should be noted that excessive doses of Sal B may have the opposite effect. Elucidating the targets of Sal B and its correlation between the regulatory effect on oxidative stress and homeostasis of energy metabolism will be helpful to further understanding its pharmacological effects under different conditions, thereby providing a scientific basis for its further clinical research and application.

## Author Contributions

ZX, JC, and PL wrote the manuscript and were involved with project concept and submission; ZX, WL, YM, HZ, XW and CZ performed data collection; JC and PL revised the manuscript and were responsible for final approval; all authors contributed to this manuscript.

## Funding

This work is supported by National Natural Science Foundation of China (No.81530101, No.81973613 and 81673780), and supported by Shanghai Rising-Star Program (19QA1408900).

## Conflict of Interest

The authors declare that the research was conducted in the absence of any commercial or financial relationships that could be construed as a potential conflict of interest.

## Abbreviations

**Abbreviations:** ROS, Reactive oxygen species; Sal B, Salvianolic acid B; SIRT1, Sirtuin 1; SOD, Superoxide dismutase; TLR, Toll-like receptor; TNF, Tumor necrosis factor; NADH, Nicotinamide adenine dinucleotide; NADPH, Nicotinamide adenine dinucleotide phosphate; NOX, NADPH oxidase; NOS, Nitric oxide synthase; COX, Cyclooxygenase; NF-κB, Nuclear factor κB; Nrf2, Nuclear factor E2-related factor 2; GSH-Px, Glutathione peroxidase; HO-1, Heme oxygenase-1; Grx1, Gluredoxin 1; Keap1, Kelch-like ECH-related protein 1; PI3K, Phosphatidylinositol 3-kinase; PARP-1, Poly (ADP-ribozyme) polymerase 1; LPS, Lipopolysaccharide; MAPK, Mitogen-activated protein kinase; HSCs, Hepatic stellate cells; VEGF, Vascular endothelial growth factor.
